# Different Quaternary Structures of Human RECQ1 Are Associated with Its Dual Enzymatic Activity

**DOI:** 10.1371/journal.pbio.0050020

**Published:** 2007-01-16

**Authors:** Laura Muzzolini, Fabienne Beuron, Ardan Patwardhan, Venkateswarlu Popuri, Sheng Cui, Benedetta Niccolini, Mathieu Rappas, Paul S Freemont, Alessandro Vindigni

**Affiliations:** 1 International Centre for Genetic Engineering and Biotechnology, Trieste, Italy; 2 Division of Molecular Biosciences, Faculty of Natural Sciences, Imperial College London, London, United Kingdom; National Cancer Institute–National Institutes of Health, United States of America

## Abstract

RecQ helicases are essential for the maintenance of chromosome stability. In addition to DNA unwinding, some RecQ enzymes have an intrinsic DNA strand annealing activity. The function of this dual enzymatic activity and the mechanism that regulates it is, however, unknown. Here, we describe two quaternary forms of the human RECQ1 helicase, higher-order oligomers consistent with pentamers or hexamers, and smaller oligomers consistent with monomers or dimers. Size exclusion chromatography and transmission electron microscopy show that the equilibrium between the two assembly states is affected by single-stranded DNA (ssDNA) and ATP binding, where ATP or ATPγS favors the smaller oligomeric form. Our three-dimensional electron microscopy reconstructions of human RECQ1 reveal a complex cage-like structure of approximately 120 Å × 130 Å with a central pore. This oligomeric structure is stabilized under conditions in which RECQ1 is proficient in strand annealing. In contrast, competition experiments with the ATPase-deficient K119R and E220Q mutants indicate that RECQ1 monomers, or tight binding dimers, are required for DNA unwinding. Collectively, our findings suggest that higher-order oligomers are associated with DNA strand annealing, and lower-order oligomers with DNA unwinding.

## Author Summary

The transient opening of the DNA double helix is a fundamental step in several DNA metabolic processes. This reaction is driven by proteins called helicases, which make use of ATP as fuel to unwind the DNA duplex. The RecQ family of helicases help maintain genome stability. Recent studies have shown that RecQ helicases, in addition to promoting DNA unwinding, can also catalyze the opposite reaction—the pairing of the partially unwound DNA duplexes. The mechanisms underlying the regulation of this dual enzymatic activity are, however, unknown. Here we describe two structural forms of the human RECQ1 helicase, a large oligomeric complex composed of five or six subunits, and a smaller form consistent with only one or two molecules. We provide an initial view of the three-dimensional structure of the larger complex and show that this state is associated with DNA strand annealing, whereas the smaller form carries out DNA unwinding. The functional switch from strand-annealing to DNA unwinding is controlled by ATP binding, which promotes the dissociation of the larger, higher-order complexes. By providing insight into the mechanisms regulating RecQ helicase activity, our study opens a new window onto a fundamental aspect of DNA metabolism.

## Abstract 3

RecQ helicases can either unwind or anneal strands of DNA. This dual functionality is explained by electron microscopy studies; higher-order oligomers appear responsible for annealing, whereas lower-order oligomers control DNA unwinding.

## Introduction

The transient opening of nucleic acid duplexes is a key step in many DNA and RNA metabolic processes. This reaction is catalyzed by helicases, a rapidly growing family of enzymes that unwind nucleic acids in a reaction coupled to the binding and hydrolysis of nucleoside triphosphate (NTP) [1–3]. A feature common to all the proposed unwinding mechanisms is the necessity for helicases to contain multiple DNA or RNA binding sites in order to unwind the duplex and translocate processively without dissociating from the nucleic acid lattice [4,5]. Multiple DNA binding sites can be present within a monomer or can be provided by separate subunits of an oligomeric complex. Thus, the determination of the active oligomeric structure of a helicase is the first step toward understanding the mechanism of DNA unwinding. Some helicases, such as PcrA from *Bacillus stearothermophilus,* function as monomers, and an “inchworm” unwinding model has been proposed in which the enzyme possesses two non-identical DNA binding sites that simultaneously bind single-stranded DNA (ssDNA) and double-stranded DNA (dsDNA) [6,7]. In contrast, a different model has been suggested for the Escherichia coli Rep and UvrD helicases in which the two subunits of a dimeric complex are required to promote strand separation [8,9]. Furthermore, several replicative helicases, such as the bacteriophage T4 gp41 and the SV40 large T antigen proteins, have been shown to assemble into ring-shaped hexamers that might encircle the DNA resulting in high processivity [10–12].

RecQ enzymes are a class of helicases found in a variety of organisms, including both prokaryotes and eukaryotes, that play an essential role in the maintenance of chromosome stability [13,14]. Five RecQ helicases have been found in humans: RECQ1 (alias RECQL), RECQ4, RECQ5, BLM, and WRN. Mutations in the genes of RECQ4, BLM, and WRN are responsible for three genetic disorders associated with inherent genomic instability and cancer predisposition [15–17]. Biochemical studies have demonstrated that RecQ helicases unwind DNA with a 3′ to 5′ polarity and, although with some differences, are capable of unwinding a variety of DNA structures other than standard B-form DNA duplexes such as forked duplexes, D-loops, triple helices, 3- or 4-way junctions, and G-quadruplex DNA [18–22]. In addition to unwinding DNA, various studies have shown that RECQ1, BLM, WRN, RECQ4, and RECQ5 catalyze strand annealing between complementary ssDNA fragments in a reaction that is modulated by ATP binding [23–27]. The mechanism that regulates the balance between the dual antagonistic activities of RecQ helicases is, however, still unknown, and the information available on the oligomeric structure of RecQ helicases in the presence and absence of ATP and/or DNA is limited and, in some cases, contradictory [14].

Assembly states ranging from monomers to hexamers have been previously described for various RecQ enzymes. Electron microscopy and size exclusion chromatography experiments have shown that the full-length BLM protein forms ring-like oligomers in the absence of ATP and DNA [28]. However, recent studies of the deletion mutant BLM^642-1290^ revealed that it retains helicase activity and runs as a monomer on a gel filtration column, both in solution and in its ssDNA-bound form, suggesting that monomers are responsible for DNA unwinding [29]. Size exclusion chromatography and dynamic light scattering studies suggested that RECQ1 forms dimers in solution [30], whereas similar size exclusion experiments indicated that full-length recombinant WRN as well as WRN^1–333^ elute as trimers [31]. Subsequent atomic force microscopy analysis of the 171–amino acid fragment of WRN comprising the exonuclease activity of the enzyme revealed a trimer–hexamer equilibrium in the absence of DNA and that this equilibrium is significantly shifted toward the hexamer in the presence of a 3′-recessed dsDNA molecule [32]. The functional role of these different oligomeric forms is, however, still unknown and the subject of controversy. The recent discovery that BLM, WRN, and RECQ1 have two opposite enzymatic activities prompted us to investigate whether different assembly states of RECQ1 are linked to DNA unwinding and strand annealing.

The electron microscopy and biochemical studies described here provide the first direct insight into the mechanism that controls the dual function of RECQ1. Our results demonstrate that RECQ1 forms higher-order oligomeric structures in solution in the presence of ssDNA and suggest that an ATP-induced change in RECQ1 oligomeric state is associated with the switch from the DNA unwinding to the strand annealing mode.

## Results

**Figure 1:**
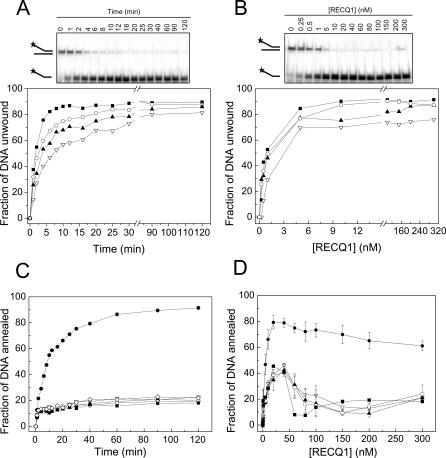
Analysis of the Unwinding and Strand Annealing Activities of RECQ1 (A) Kinetics of unwinding performed using 5 nM RECQ1. (B) RECQ1 unwinding at varying enzyme concentrations (15-min reaction). (C) Kinetics of strand annealing in the presence of 20 nM RECQ1 and in the absence of enzyme (open diamond [◊]). (D) Analysis of RECQ1 strand annealing activity as a function of protein concentration (15-min reaction). Data points were the mean of three independent experiments with the standard deviation indicated by error bars. The concentration of ATP in all the experiments varied from 0 to 5 mM (filled circle [•] = 0 mM, open inverted triangle [▿] = 0.5 mM, filled triangle [▴] = 1 mM, open circle [○] = 2 mM, and filled square [▪] = 5 mM).

The 649–amino acid variant of RECQ1 was expressed in baculovirus with high yield following a previously described procedure [30]. Unwinding experiments using a forked duplex substrate with 3′ and 5′ single-stranded arms showed that 86% of the substrate was unwound within 15 min using 5 nM enzyme (Figure 1A). Previous studies indicated that BLM can also unwind more than 80% of a forked DNA duplex under similar reaction conditions [23]. However, the level of ssDNA product declined significantly at protein concentrations higher than 10 nM, suggesting that BLM promotes re-annealing of the two strands at high protein concentration. In contrast, our concentration-dependence experiments showed that the fraction of substrate unwound remained constant at increasing [RECQ1], indicating possible differences between the enzymatic activities of BLM and RECQ1 (Figure 1B). Kinetic and concentration-dependence experiments showed, however, that RECQ1 was able to promote efficient strand annealing in the absence of ATP (Figure 1C and 1D).

The addition of ATP or the poorly hydrolysable analog ATPγS strongly inhibited the strand annealing reaction (Figures 1C, 1D, and see below), whereas ADP had no significant effect (unpublished data), in agreement with previous findings [27]. Moreover, ATP concentration-dependence studies indicated that, in the case of the 20–base pair (bp) forked duplex probe, the balance between the unwinding and strand annealing activity of RECQ1 was markedly shifted toward unwinding even in the presence of low ATP concentrations (Figure 1). The strand annealing inhibition was not due, however, to a competition between the helicase and strand annealing activities of the protein, because analogous results were obtained using a blunt-ended probe that cannot be unwound by RECQ1 in the presence of ATP (see below). Our previous limited proteolysis experiments performed in the presence and absence of the nucleotide showed that the addition of ATP modified the chymotrypsin digestion pattern of RECQ1, rendering the protein more resistant to proteolysis [27]. This result suggests that ATP binding either protects RECQ1 from proteolytic digestion by covering some cleavage sites or it induces a conformational change in RECQ1 associated with the switch of RECQ1 from its strand annealing to its DNA unwinding mode.

### Size Exclusion Chromatography

**Figure 2:**
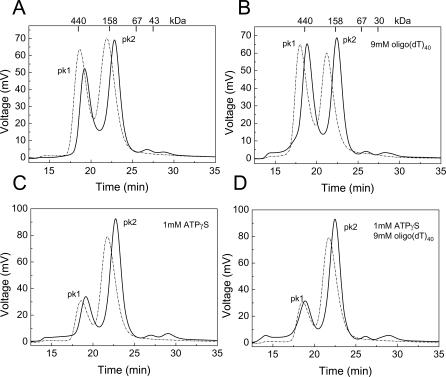
Size Exclusion Chromatography Experiments Chromatographic profiles of the untagged RECQ1 (solid lane) and His_6_-RECQ1 (dashed line) eluting from the Superdex200 HR 10/30 gel filtration column. The protein species were detected by protein fluorescence (λ_excitation_ = 290 nm and λ_emission_ = 340 nm). Approximately 40 μg of recombinant RECQ1 were loaded at a final concentration of 1 μM. The protein eluted in two main peaks. The first peak corresponds to a calculated molecular mass of approximately 400 kDa, whereas the second corresponds to a calculated molecular weight of approximately 155 kDa. The absence of protein eluting with the void volume highlights the lack of nonspecific aggregates. (A) RECQ1 alone. (B) RECQ1 + ssDNA (oligo(dT)_40_). (C) RECQ1 + ATPγS. (D) RECQ1 + ssDNA (oligo(dT)_40_) + ATPγS. Analogous results were obtained using oligo(dT)_30_ (unpublished data).

Size exclusion chromatography experiments in the presence and absence of ATP and/or ssDNA were performed to analyze the molecular basis of the change in conformation and/or oligomeric state associated with nucleotide binding. Our previous gel filtration studies showed that RECQ1 eluted as a protein with an apparent molecular mass (Mr) of 158 kDa, suggesting that RECQ1 is a dimer in solution in the absence of ATP and ssDNA [30], although the possibility that RECQ1 is a monomer that elutes earlier than expected because of its elongated structure could not be ruled out. New size exclusion chromatography experiments performed in buffer containing a 50 mM higher salt concentration confirmed the presence of a “dimeric” form of RECQ1 (peak 2), but also showed a second peak eluting earlier (peak 1) (Figure 2). This early eluting peak had an apparent Mr of approximately 400 kDa, falling between the calculated Mr of a RECQ1 pentamer (365 kDa) and hexamer (438 kDa). Further experiments using salt concentrations ranging from 100 to 500 mM confirmed that the ratio of the two peaks was significantly affected by salt concentration, with high salt favoring the early eluting peak, suggesting that hydrophobic interactions may be involved in higher-order oligomer formation (Figure 2 and unpublished data). Experiments at different protein concentrations indicated that higher protein concentrations again increased the ratio between peak 1 and peak 2 (Figure S1). Interestingly, the addition of ssDNA (oligo(dT)_40_ or oligo(dT)_30_) also favored the early eluting peak, whereas the presence of ATP or ATPγS significantly shifted the equilibrium toward the smaller oligomeric form both in the presence and absence of ssDNA (Figure 2). The same results were obtained after removal of the His_6_-tag at the N-terminus of RECQ1 (utRECQ1), thus excluding any possible contribution of the tag in promoting oligomerization (Figure 2), which is consistent with the His_6_-tagged and untagged RECQ1 having almost identical ATPase, unwinding, and strand annealing activities (Figure S2). Furthermore, gel filtration experiments repeated in the presence and absence of Mg^2+^ indicated that divalent cations do not affect the oligomerization state of RECQ1 (unpublished data). In conclusion, our size exclusion chromatography results suggest that RECQ1 can exist under different oligomeric forms and that higher-order oligomers are destabilized by nucleotide binding.

### Single-Particle Electron Microscopy and Three-Dimensional Reconstruction

To obtain more detailed information on the oligomeric structure of RECQ1, we performed single-particle negative stain, transmission electron microscopy (TEM) experiments in the presence and absence of ATP and/or ssDNA. One set of experiments with untagged RECQ1 alone was performed under the same conditions as the gel filtration studies and revealed the presence of a mixed population of particles. We observed particles with sizes of approximately 35 Å and 55 Å, and ring-like structures of 100 Å, which could be consistent with the presence of monomers, dimers, and larger oligomeric structures, respectively (Figure S3A). Although we cannot distinguish between monomers of RECQ1 and different possible views of RECQ1 dimers, the addition of an oligo(dT)_30_ promoted the formation of a more homogeneous population enriched in the same approximately 100 Å–diameter oligomeric ring-like particles (Figure S3B). This suggests that although RECQ1 might already exist in different quaternary forms after expression and purification, ssDNA appears to stabilize larger oligomeric structures, consistent with our gel filtration data. Strikingly, the addition of ATP or its slowly hydrolysable analog ATPγS resulted in the almost complete disruption of these oligomers whether ssDNA was present or not. His-tagged RECQ1 examined by TEM also exhibited a similar behavior to utRECQ1 in that larger oligomeric structures were observed in the presence of ssDNA that were lost upon the addition of ATP (unpublished data).

**Figure 3:**
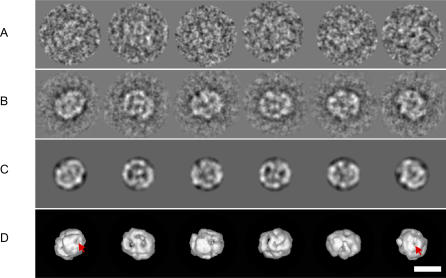
Image Processing and 3D Reconstructions of RECQ1 Oligomers Representative images from the various stages of image-processing procedures using negatively stained untagged RECQ1 + ssDNA sample as an example. (A) Examples of individual particle images after band-pass filtering and alignment. These images are members of the corresponding class averages shown in the row below (protein is white). (B) Class averages obtained after classification. (C) Re-projections of the 3D density map in orientations corresponding to the Euler angles assigned to the class averages in the row above. (D) Surface renderings of the density map at a threshold level of σ = 3 in orientations corresponding to the Euler angles assigned to the class averages in (B). The left- and right-most images correspond to approximately diametrically opposite views of the molecule, whereas the images in-between correspond to views that are rotated by approximately 90° with respect to these, and also with respect to each other. Scale bar represents 10 nm.

Image processing of various RECQ1 samples revealed that the particles adopted different orientations on the carbon supporting film making them amenable to three-dimensional (3D) reconstruction by angular reconstitution methods. The initial eigenimage analysis based on translational alignment revealed, in some cases, a few eigenimages with apparent 2-fold or 6-fold rotational symmetry (unpublished data). However, upon closer examination, it became clear that the symmetry was not strict, and, moreover, an analysis of the resulting class-average images did not reveal any strongly symmetric projections. As a consequence, all image processing for the RECQ1 datasets was performed without imposing symmetry. Different steps in the 3D reconstruction procedure performed on the RECQ1 protein in the presence of ssDNA are shown in Figure 3. Representative class averages (Figure 3B) are presented with the corresponding re-projection (Figure 3C) of the 3D map in the direction of the assigned Euler angles. The good agreement between these images is a visual estimation of the quality of the reconstruction. We observe that the protein density is arranged around two stain-accumulated pores in both head-on views of the structure (Figure 3D, red arrows) indicating a possible straight conduit running through the length of the helicase. Identical results were obtained using the His_6_-RECQ1 in the presence of ssDNA.

**Figure 4:**
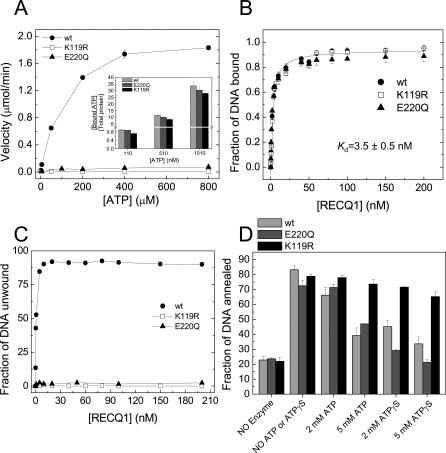
Characterization of the Enzymatic Activity of the K119R and E220Q Mutants (A) ATPase assays for wild-type (wt) RECQ1 and its mutants. Thin-layer chromatography assays were performed using M13 ssDNA (32 μM) and 20 nM RECQ1 in buffer A as previously described [30]. The plots are the average of three independent experiments. The insert shows the ATP binding assays for wild-type, K119R, and E220Q proteins performed by nitrocellulose filter binding at three different ATP concentrations. (B) DNA binding assay at increasing protein concentrations using the forked duplex probe. EMSA experiments were performed in buffer 20 mM Tris-HCl (pH 7.5), 50 mM NaCl, 2 mM MgCl_2_, 1 mM DTT, and 0.1 mg/ml of BSA as previously described [30]. The plots are the average of three independent experiments. (C) Unwinding assay at varying enzyme concentrations. The fractions of protein-dependent unwound product formation for the wild-type RECQ1 and its mutants are plotted. (D) Strand annealing assays with two fully complementary oligonucleotides that form a blunt-ended duplex of 42 bp upon annealing. The reaction was carried out for 15 min at 37 °C using 20 nM wild-type, K119R, or E220Q plus or minus the indicated concentrations of ATP or ATPγS. The plots are the average of three independent experiments with the standard deviation indicated by error bars.

The structure of the RECQ1 K119R mutant, in which the invariant lysine residue (K119) in the conserved ATPase motif I (Walker A box) was replaced with Arg, was also analyzed by electron microscopy (EM). The RECQ1(K119R) mutant is deficient in ATP hydrolysis and, interestingly, yielded a more homogenous population of particles allowing a higher-resolution reconstruction to be obtained (see below). This mutant retained the ability of ATP and ssDNA binding, although its affinity for ATP was slightly reduced relative to wild-type RECQ1 and did not show any detectable ATPase or unwinding activity (Figure 4). The strand annealing activity of RECQ1(K119R) was comparable to that of the wild-type protein, even though, unlike the wild type, the annealing reaction was not inhibited by the addition of increasing concentrations of ATP or ATPγS (Figure 4). The initial two-dimensional EM averages of RECQ1(K119R) showed that this mutant also formed larger oligomeric structures in the presence of ssDNA, similar to wild-type RECQ1. Interestingly, similar oligomeric particles were still present for the K119R mutant upon addition of ATPγS, suggesting that binding of nucleotide to this variant does not disrupt these pre-formed oligomeric structures, unlike the wild-type protein. These results are supported by gel filtration studies showing that the ratio between the early and late eluting peaks of K119R is not affected by the addition of ATP, in contrast to what was previously observed for the wild-type protein (Figure S4).

**Figure 5:**
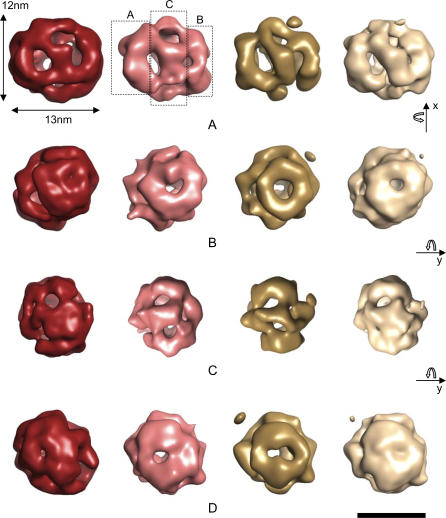
Surface Rendering of 3D EM Reconstructions of RECQ1 Oligomers Untagged wild-type RECQ1 + ssDNA (red); wild-type RECQ1+ssDNA (pink); K119R mutant RECQ1 + ATPγS (gold); and K119R mutant RECQ1 + ssDNA (white). All maps are shown using an arbitrary density threshold level of 3σ. The general shape of RECQ1 in the four functional states shows an overall similarity and consists of two ring-like densities “A” and “B,” interconnected by a larger ring-like structure “C.” (A) Side-on views showing the basic three-ring structure with the middle ring “C” connecting the two outer rings with diagonally spanning densities. (B) Head-on views of ring “A,” which has an outer diameter of 8 nm. (C) Side-on views rotated by 90° about the *y*-axis relative to (B). (D) Head-on views of ring “B,” which has approximately the same outer diameter as ring “A.” The resolutions of the maps range from 22 Å for the tagged RECQ1 with ssDNA reconstruction to approximately 26 Å for the untagged RECQ1 with ssDNA reconstruction. Scale bar represents 10 nm [56].

Despite the different sample conditions and resolutions between the EM maps, all the RECQ1 samples, including the wild type (untagged or tagged) plus ssDNA and the K119R mutant plus ssDNA or ATPγS, revealed a cage-like structure of approximately 120 Å × 130 Å with a complex organization (Figure 5). The resolution of the maps as measured by Fourier Shell Correlation (FSC) and the 1/2-bit threshold criterion ranged from 22 Å for the tagged RECQ1 with ssDNA reconstruction to approximately 26 Å for the untagged RECQ1 with ssDNA reconstruction. Preliminary modeling of the crystal structure of the catalytic domain of the E. coli RecQ into the EM maps shows that either a pentamer- or hexamer-like RecQ structure can be accommodated within the density consistent with the gel filtration studies. However, higher-resolution cryo-EM studies would be needed to unambiguously determine the RECQ1 oligomeric packing arrangement. The body of the helicase is arranged as three ring-like domains connected by bridges of density (Figure 5A). Two planar rings (or “wheels”) are flanking both sides of the central core, which is made of slanted densities, giving the appearance of a spiral. The side pores are visible in Figure 5B and 5D although there appears to be some variation in their size when DNA is present. At the other end of the RECQ1 oligomer, six side densities converge around a small central pore, whereas two sides of the oligomer exhibit a wheel shape with a central pore of approximately 20 Å in diameter (Figure 5B).

### Competition Assay

**Figure 6:**
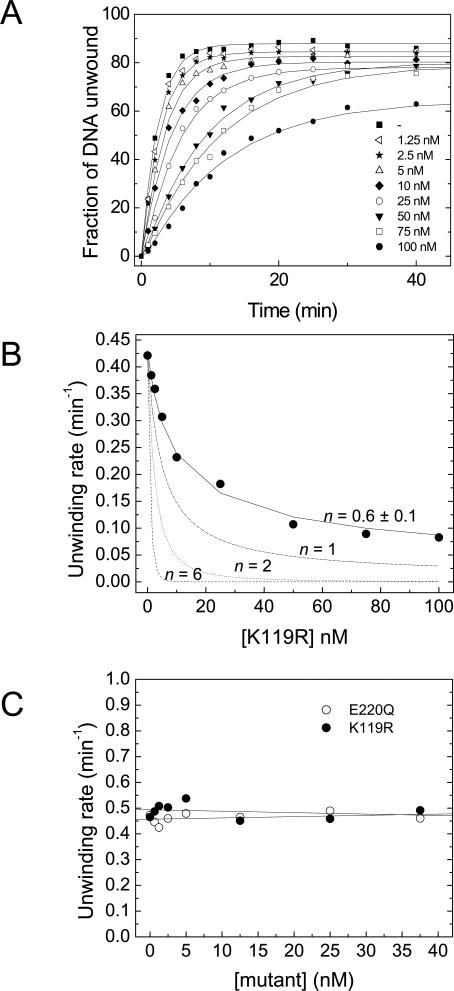
Competition Experiments with the K119R and E220Q Mutants (A) Time courses of the unwinding reactions with 5 nM RECQ1 containing 0–100 nM K119R. The fraction of ssDNA released as a product of the unwinding reaction is plotted versus time. (B) Plot of the unwinding rate versus the concentration of K119R. The solid line represents the fit of the experimental data to Equation 1 with *n* = 0.6 ± 0.1. The dashed-dotted, dashed, and dotted lines simulate the expected variation of unwinding rate for *n* = 1, 2, or 6. (C) Rates of unwinding by wild-type RECQ1 in the presence of increasing concentrations of K119R (filled circle [•]) and E220Q (open circle [○]) under single-turnover conditions. A total of 50 nM oligo(dT)_40_ is utilized to trap the helicase that dissociates from the substrate during the reaction.

To learn more about the oligomeric structure of RECQ1 responsible for DNA unwinding, we performed competition experiments using the K119R RECQ1 mutant that has no detectable helicase activity. Electrophoresis mobility shift assay (EMSA) experiments indicated that this single–amino acid substitution did not affect DNA binding. In fact, binding isotherms obtained at increasing protein concentrations showed that wild-type and mutant RECQ1 bound the forked duplex probe with equal affinity and with an apparent *K*_d_ value of 3.5 ± 0.5 nM (Figure 4B). We then performed a series of unwinding experiments using a fixed concentration of wild-type RECQ1 (5 nM) and increasing concentrations of the mutant protein. In order to provide equal binding opportunity for both proteins, wild-type and mutant RECQ1 were premixed prior to adding the forked duplex DNA substrate to the helicase reaction, and unwinding was initiated upon the addition of 5 mM ATP. The presence of increasing concentrations of the K119R mutant caused a mild decrease in the rate of unwinding (Figure 6A). Analogous results were obtained when the reaction was initiated by ssDNA instead of ATP (unpublished data). The observed decrease in the unwinding rate can be due to competition between the wild-type and mutant protein for substrate binding, or to the formation of hetero-oligomers between wild-type monomers and mutant monomers that unwind the substrate at a slower rate. Data analysis under the assumption that only homo-wild-type complexes are capable of unwinding at a maximal rate (Equation 1) yielded a value of *n* = 0.6 ± 0.1, where *n* indicates the number of subunits forming the RECQ1 complex [33]. Competition assays were then performed using forked duplexes of 30 and 40 bp to see if the length of the duplex affects the results, given that partially unwound duplexes can spontaneously melt in the presence of an active helicase [34]. The length of the duplex utilized in the assays, however, did not affect the value of *n* obtained from the fit (Figure S5). Simulations for values of *n* = 2 or 6 completely failed to fit the data, suggesting that the RECQ1 helicase does not form hetero-oligomers (Figure 6B). However, simulation curves obtained for *n* = 1 did not properly fit our experimental data either, suggesting that other factors might complicate the interpretation of these experiments.

Thus, we repeated the kinetic experiments with the premixed proteins in the presence of a 100-fold excess of a non-specific ssDNA trap that was added to the reaction mix in order to prevent RECQ1 from reassociating to the forked DNA substrate. Under these single-turnover conditions, the specific activity of the wild-type RECQ1 helicase should not be affected by the addition of the mutant if hetero-oligomers do not participate at the unwinding reaction. In agreement with this hypothesis, we found that the unwinding rate of RECQ1 did not change by increasing the concentration of the K119R protein under conditions in which only a single cycle of unwinding was observed (Figure 6C). To demonstrate that the inability of the K119R mutant to affect wild-type RECQ1 helicase activity was not due to the intrinsic structural properties of the K119R protein, the same experiments were performed using an additional RECQ1 mutant (E220Q) in which the Glu residue of the Walker B domain was replaced with Gln. Previous studies have shown that this residue coordinates the interaction with a water molecule required for ATP hydrolysis [35–37], and its substitution to Gln does not affect ATP binding [38–40]. Consistently, our results showed that the E220Q ATPase dead mutant bound ATP with the same affinity as the wild-type protein (Figure 4A). Moreover, its strand annealing activity was inhibited by the addition of ATP or ATPγS, as previously observed for the wild-type protein (Figure 4D). Single-turnover experiments performed with the E220Q mutant also confirmed the results obtained with the K119R protein, supporting our conclusion that hetero-oligomers are not involved in the unwinding reaction and suggesting that the functional unit responsible for DNA unwinding is either a monomer or a tight-binding dimer that cannot mix with ATPase-deficient mutants.

## Discussion

RecQ helicases are an emerging family of enzymes that play a key role in the maintenance of chromosome stability at the interface between DNA replication and repair [13,14,41]. All DNA helicases need to posses at least two DNA binding sites in order to simultaneously travel along the DNA lattice and transiently open duplexes [3,4,6,10,11]. Thus, information on the quaternary structure of these enzymes is essential to understand whether multiple DNA binding sites are distributed on a single molecule or on separate subunits of an oligomeric complex. Thus far, data on the oligomeric state of RecQ helicases have been limited and controversial. For instance, the crystal structure solved for the E. coli RecQ^1–516^ mutant lacking the Helicase-and-RNase-D-C-terminal (HRDC) domain indicates that its catalytic core is monomeric in its nucleotide bound and unbound forms [42]. Nevertheless, conflicting results have been reported on the oligomerization state of the full-length E. coli RecQ helicase in solution [43,44]. Similarly, studies on the human BLM, WRN, and RECQ1 helicases have reported quaternary structures that range from monomers to higher-order oligomers [28,30,31,45,46]. Thus, the question on the assembly state associated with the mechanism of DNA recognition and unwinding by RecQ helicases is still uncertain. Moreover, recent findings indicating that, in addition to unwinding DNA, RECQ1, BLM, WRN, and RECQ5 promote annealing of complementary ssDNA fragments suggest that the conformation and/or oligomeric state of these helicases may change in order to switch from a strand annealing to a DNA unwinding mode [23–25,27]. Currently, there is no information on the molecular basis for this switch or on the quaternary structure of RecQ helicases that promotes strand annealing.

Our work reports the first analysis on the oligomeric structure of the human RECQ1 helicase in the presence and absence of different nucleotides and/or ssDNA. Using size exclusion chromatography and transmission electron microscopy, we compared the assembly state of RECQ1 under conditions in which the enzyme is proficient in strand annealing versus unwinding. Efficient annealing of complementary ssDNA fragments by RECQ1, BLM, and WRN occurs only in the absence of ATP [23,25,27]. The presence of ATP or its slowly hydrolysable analog ATPγS inhibits the annealing reaction. Limited proteolysis experiments suggest that this inhibition may be due to an ATP-induced change in the conformation and/or assembly state of RECQ1 [27]. Consistently, our size exclusion chromatography experiments show that the elution profile of RECQ1 is significantly affected upon nucleotide addition. In particular, ATP or ATPγS favors the smaller oligomeric-form of RECQ1, whereas ssDNA appears to stabilize larger oligomeric states consistent with pentamers or hexamers. Our observation that ATP and ATPγS yield analogous results suggests that nucleotide binding, rather than hydrolysis, causes an equilibrium shift toward the smaller oligomeric form.

Our EM studies of recombinant RECQ1 revealed the presence of a mixed population of RECQ1 particles, consistent with monomers, dimers, and larger ring-like oligomeric structures. In agreement with the gel filtration data, larger oligomers are stabilized under conditions in which RECQ1 promotes strand annealing, whereas the addition of ATP or ATPγS results in the almost complete disruption of higher-order oligomers. Taken together, our results support a role for larger RECQ1 oligomers in strand annealing and smaller RECQ1 oligomers in DNA unwinding. In agreement with this conclusion, Hickson and co-workers found that BLM mutants lacking strand annealing activity fail to form higher-order protein–DNA complexes by gel retardation assays, suggesting that oligomerization is required for the strand annealing function of BLM [23]. Our observation that the higher-order oligomers are still present for the ATPase-deficient mutant K119R upon addition of ATP indicates that binding of nucleotide to this variant does not disrupt these pre-formed oligomeric structures, unlike the wild-type protein, and further supports the conclusion that higher-order oligomers are required for the strand annealing reaction. In fact, the strand annealing activity of K119R is not inhibited by ATP as previously observed for the wild-type protein.

Our EM reconstructions extend previous studies of human RecQ helicases [28] by providing a 3D envelope for full-length RECQ1. The striking similarity of the 3D structures obtained for the wild-type RECQ1 plus ssDNA and the K119R mutant plus ssDNA or ATPγS is an indicator of the robust nature of our EM analysis. Although the estimated volume of our EM maps, at a threshold of approximately1.5 σ, is consistent with RECQ1 hexamers, we have not been able to unambiguously determine the packing symmetry of the RECQ1 monomers. This could reflect the complex nature of the packing arrangement as well as the limitations of negative-stain EM. Nevertheless, our RECQ1 reconstructions are reminiscent of other helicases with oligomeric ring-like structures surrounding a central pore [11]. Our RECQ1 EM maps show the body of the helicase to be arranged in two ring-like densities interconnected by diagonally extending densities to a larger middle ring-like structure. The three rings seem to form a hollow channel with an inner diameter of approximately 20 Å. Although highly speculative, the central pore could accommodate dsDNA with the diagonally slatted densities aiding ssDNA to wrap around the oligomer and into the central channel where it could associate with the other strand that threads through the full length of the channel. An alternative model is that RECQ1 may promote strand annealing in a similar manner to that observed for the RAD52 recombination protein. RAD52 forms oligomeric rings with the ssDNA bound to an exposed groove on the surface of the ring, and multiple RAD52–DNA complexes associate to facilitate the paring of the bases and promote the strand annealing reaction [47]. Further higher-resolution RECQ1 structures in the presence of DNA will be required to confirm these models.

To gain more insights into the oligomeric structure of RECQ1 required for DNA unwinding, we performed competition experiments with the ATPase-deficient K119R mutant. Our initial analysis of the reduction in helicase activity as a function of increasing mutant concentrations did not provide clear information on the assembly state of RECQ1 associated with DNA unwinding. Thus, competition experiments were repeated under single-turnover conditions. Under these conditions, the helicase that dissociates from the substrate is trapped by the non-specific ssDNA added to the reaction. The fact that no trend in the rate of unwinding is observed in the presence of the ssDNA trap provides evidence that the oligomerization state of RECQ1 does not change in the presence of the K119R mutant. The same experiments performed with the E220Q mutant indicate that the lack of unwinding-rate inhibition observed with the K119R mutant is not due to the intrinsic inability of this protein to mix with the wild-type enzyme. Collectively, our competition assays suggest that hetero-oligomers do not participate in the unwinding reaction and that the functional RECQ1 unit responsible for DNA unwinding is a monomer. However, the possibility that RECQ1 forms tight-binding dimers that cannot mix with the ATPase-deficient mutants cannot be ruled out at this stage. Thus, further studies will be required to establish whether RECQ1 monomers, tight-binding dimers, or both are involved in nucleic acid unwinding. Analogous competition experiments performed with the E. coli RecQ and its ATPase-deficient K55A mutant suggested that the protein functions as a monomer [44]. More recently, kinetic studies on the E. coli enzyme reinforced this conclusion [46]. Consistently, similar experiments using the WRN helicase suggested that also for the WRN protein, a single subunit is involved in DNA unwinding [45], whereas Janscak and co-workers showed that the catalytically active deletion mutant BLM^642-1290^ runs as a monomer on a gel filtration column, both in solution and in its ssDNA-bound form [29]. On this basis, it is tempting to speculate that, although RecQ helicases are dynamic enzymes that adopt different structures when bound to different reaction components such as DNA and/or ATP, they might share a common unwinding mechanism in which smaller oligomers, possibly monomers, are required for the transient opening of the DNA duplexes.

In summary, our results provide a functional explanation for the role of the different assembly states previously described for several RecQ helicases and indicate that ATP binding is the key that triggers the switch from the strand annealing to the DNA unwinding mode. However, additional factors might be required to stabilize the different oligomeric forms of RECQ1 in vivo. For example, human replication protein A (hRPA) may stimulate RECQ1 helicase activity by binding smaller oligomers with higher affinity than higher-order oligomers. Similarly, other proteins, yet to be elucidated, may interact with RECQ1 pentamers or hexamers with higher affinity and in this way promote the strand annealing activity of RECQ1. A challenging avenue for future studies would be to test if novel factors that interact with human RecQ helicases specifically stimulate one of the two opposite enzymatic activities.

The dual enzymatic activity of RecQ helicases may be crucial for their function in vivo. In the case of human BLM, it was suggested that dual enzymatic activity is required for the formation and resolution of the so-called chicken-foot structures that are formed to bypass lesions on the leading-strand template during DNA replication [23]. Alternatively, BLM strand annealing and unwinding activities might be needed for the resolution of double Holliday junction structures that arise during recombinational repair of replication forks [48,49]. On the other hand, the biological relevance of our results cannot be clearly evaluated due to the lack of information on the phenotypes of human *RECQ1* mutant cells. Genetic complementation studies with chicken DT40 cells indicated that the double mutant RECQ1^−/−^ BLM^−/−^ cells have an elevated rate of sister chromatid exchange after mitomycin-C treatment compared to BLM^−/−^ cells, suggesting that the two proteins might have some redundant functions [50]. On the basis of the observation that RECQ1 promotes strand annealing and is able to unwind typical recombination intermediates such as D-loop structures, Sharma et al. suggested that the RECQ1 might be involved in the synthesis-dependent strand annealing (SDSA) repair pathway in which multiple cycles of unwinding and annealing are required [27]. However, future studies will be necessary in order to uncover the repair pathway that requires the coordinated action of the unwinding and strand annealing activity of RECQ1.

## Materials and Methods

### Proteins.

The RECQ1-K119R construct was generated with the QuickChange XL site-directed mutagenesis kit (Strategene, La Jolla, California, United States) using the same protocol previously described for the RECQ1-K119A mutant [51]. The wild-type and mutant proteins were purified from baculovirus/Sf9 cells as already described [30].

### Sequence of DNA substrates.

The fork and blunt-ended duplex DNA substrates were made using the polynucleotides: 5′CTTGCTTGTGTAGCCCATGCTTTTTTTTTTTTTTTTTTTTTTTTTTTTTT3′ and 5′TTTTTTTTTTTTTTTTTTTTTTTTTTTTTTCGTACCCGATGTGTTCGTTC3′ (20-bp fork); 5′AGTCTTCGTCCTCGTACCCGATGTTTTCGCTTTTTTTTTTTTTTTTTTTTTTTTTTTTTT3′ and 5′TTTTTTTTTTTTTTTTTTTTTTTTTTTTTTACGAAAACATCGGGTAC GAGGACGAAGACT3′ (30-bp fork); 5′TGTCGTGGTCAGTCTTCGTCCTCGT ACCCGATGTTTTCGCTTTTTTTTTTTTTTTTTTTTTTTTTTTTTT3′ and 5′TTTTTTT TTTTTTTTTTTTTTTTTTTTTTTACGAAAACATCGGGTACGAGGACGAAGACTGACCACGACA3′ (40-bp fork); 5′TGTCGTGGTCAGTCTTCGTCCTCGTACCCGATGTTTTCGTTC3′ and 3′ACAGCACCAGTCAGAAGCAGGAGCATGGGCTACAAAAGC AAG5′ (blunt-ended).

### DNA helicase assays.

The helicase assay were performed using a forked duplex DNA substrate with a 20-bp duplex region and two oligo(dT)_30_ tails. The 20-μl reaction mixture contained buffer A (20 mM Tris-HCl [pH 7.5], 8 mM DTT, 5 mM MgCl_2_, 10 mM KCl, 10% glycerol, 80-μg/ml bovine serum albumin [BSA]), 5 mM ATP, and ^32^P-labeled helicase substrate (0.5 nM). Recombinant RECQ1 was added to the mixture and incubated at 37 °C for the times specified in Figure 1. The reaction was terminated upon addition of 20 μl of 0.6% SDS, 35 mM EDTA, 25% glycerol, 0.04% bromphenol blue, and 0.04% xylene cyanol (Stop Buffer). Reaction products were resolved on a 12% native PAGE, and the extent of unwinding was quantified as previously described [30].

In the competition experiments with the K119R mutant, the unwinding activity of wild-type RECQ1 (5 nM) was measured in the presence of increasing mutant protein concentration. The wild-type and mutant proteins were pre-incubated for 20 min at 25 °C in buffer A, followed by incubation with the ^32^P-labeled helicase substrate for an additional 5 min on ice. The reaction was initiated upon addition of 5 mM ATP and incubated at 37 °C for the time specified in Figure 6. The experimental data were analyzed with the following equation [33]:

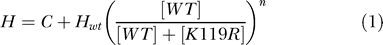
where *H_wt_* indicates the unwinding rate of the wild-type protein, *C* is the unwinding rate of hetero-oligomers, whereas [*WT*] and [*K119R*] are the concentrations of the wild-type and mutant RECQ1, respectively. The value of *n* indicates the number of subunits forming the RECQ1 complex. For the single-turnover kinetics, wild-type RECQ1 (2.5 nM) was preincubated with increasing concentrations of K119R for 20 min at 25 °C in buffer A, followed by incubation with the ^32^P-labeled fork substrate for an additional 10 min at 25 °C. The reaction was initiated upon addition of 5 mM ATP and, after 30 s, 50 nM oligo(dT)_40_ was added to trap the helicase that dissociated from the substrate during the reaction.

### DNA strand annealing assays.

The strand-annealing activity of wild-type and mutant RECQ1 was measured using partially or fully complementary synthetic oligonucleotides (0.5 nM). Strand annealing reactions (20 μl) were carried out at 37 °C in buffer A and were initiated by adding the unlabeled DNA strand. Reactions were terminated by the addition of 20 μl of Stop Buffer. Reaction products were subsequently resolved on native 12% PAGE. Radiolabeled DNA species were visualized using a Cyclone Phosphorimager (PerkinElmer Life Science, Wellesley, Massachusetts, United States) and the included OptiQuant software.

### ATP binding assays.

ATP binding was measured by filter binding on a Schleicher and Schüell 96-filter apparatus (Whatman; Brentford, United Kingdom), as previously described [52]. Nitrocellulose membrane (PROTRAN BA85 0.45 μm) was briefly soaked in 0.4 M KOH, extensively washed with distilled water, and equilibrated for 30 min at 4 °C in buffer A. The binding reactions (70 μl), containing 4 μg of protein and a fixed amount of [^32^P] γ-ATP (10 nM, 2 μCi) mixed with increasing amounts of unlabeled nucleotide in buffer A, were incubated for 20 min at 25 °C. After the incubation, 30 μl of reaction mix were applied to two different wells, filtered, and then washed three times with 500 μl of buffer A. The membrane was then air dried, exposed to a Storage Phosphor Screen (PerkinElmer Life Science, Wellesley, Massachusetts, United States) and analyzed with the OptiQuant software. BSA (4 μg) was used as control.

### Size exclusion chromatography.

Gel filtration experiments were performed on a Hewlett Packard Series 1100 HPLC system (Agilent, Santa Clara, California, United States) using a 10/30 Superdex200 HR gel filtration column (Amersham Biosciences, Munich, Germany). The column was equilibrated at a flow rate of 0.5 ml/min with 20 mM Tris (pH 7.5), 150 mM KCl, and 1 mM DTT. When specified, 1 mM ATP or ATPγS was added to the elution buffer. Approximately 40 μg of recombinant RECQ1 were loaded at a final concentration of 1 μM. Proteins were detected using a scanning fluorescence detector (λ_excitation_ = 290 nm and λ_emission_ = 340 nm). The experiments in the presence of ssDNA were performed by preincubating RECQ1 (3 μM) with 3-fold molar excess of oligo(dT)_40_ (9 μM) with or without 1 mM nucleotide for 20 min at 25 °C. Calibration was performed with the two sets of pre-mixed standard proteins of known molecular mass (Gel Filtration Calibration Kits HMW and LMW; Amersham Bioscences) as previously described [30].

### EM.

RECQ1 or RECQ1(K119R) was diluted to a concentration of approximately 15 μg/ml in 20 mM TrisHCl (pH 7.4), 150 mM KCl, 2 mM MgCl_2_, adsorbed onto a glow-discharged carbon-coated grid, and negatively stained with 1% uranyl acetate. When specified, RECQ1 was incubated with a 1.5 molar excess oligo(dT)_30_ and/or 2–5 mM nucleotide for 15 min. The grids were observed using a CM200 electron microscope operating at 200 kV. The micrographs were recorded at 50,000×, and the negatives were subsequently digitized using a CoolScan 8000 Nikon scanner (Tokyo, Japan) to a final sampling of 2.54 Å/pixel.

### Single-particle analysis and 3D reconstruction.

All image processing was carried out using the Imagic 5 software [53]. Negatively stained RECQ1 and RECQ1(K119R) particles were interactively selected from several digitized micrographs. The single particles were then iteratively centered by translational alignment relative to the rotationally averaged total sum of all images. The aligned dataset was then subjected to a multivariate statistical analysis (MSA) followed by a hierarchical ascendant classification which generated a first set of reference-free, rotationally unaligned class averages and eigenimages [54]. The resulting eigenimages were inspected for symmetry information, and a first set of references was selected after visual inspection of initial class averages. The selected views were used as references for a cycle of multi-reference alignment (MRA) followed by MSA classification. Several cycles of MRA/MSA classification were carried out until good and stable class averages were obtained. For the dataset yielding the most stable class averages (RECQ1(K119R) with ATPγS), we assigned a priori Euler angles to a six-member subset of the class averages that represented the most characteristic views, and the 3D distribution of molecular densities was reconstructed using the exact-filter back-projection algorithm and without imposing symmetry. This first raw 3D map was then refined on a small selection of classes and re-projected. The resulting re-projections were used as an anchor set for the Euler angles assignment of a larger selection of views by angular reconstitution [55], followed by cycles of MRA, MSA, classification, angular reconstitution, and back projection. For the rest of the datasets, the procedure was identical except for the fact that the initial Euler angles were not assigned a priori, but by using an anchor set based on a refined reconstruction of the RECQ1(K119R) with ATPγS dataset. A total of 1,896 particle images classified into 180 classes was used for the untagged RECQ1 with ssDNA dataset, 1,017 particle images classified into 110 classes for the tagged RECQ1 with ssDNA dataset, 1,163 particle images classified into 116 classes for the RECQ1(K119R) with ssDNA dataset, and 2,736 particle images classified into 270 classes for the RECQ1(K119R) with ATPγS dataset, to generate the final 3D models presented. The resolution of the model was assessed using the Fourier Shell Correlation (FSC) criterion [56].

## Supporting Information

Figure S1 Concentration Dependence Analysis His-tagged RECQ1 was loaded on the Superdex200 HR 10/30 gel filtration column at different concentrations in the absence of nucleotide or ssDNA. (A) 0.1 μM RECQ1, (B) 1 μM RECQ1, and (C) 35 μM RECQ1. The absence of protein eluting at the void volume highlights the lack of nonspecific aggregates.
(1.2 MB TIF)

Figure S2 Comparison of the ATPase, Unwinding, and Strand Annealing Activity of the His_6_-RECQ1 versus Untagged-RECQ1 Filled and empty symbols refer to untagged-RECQ1 and His_6_-RECQ1, respectively.
(A) ATPase assays performed with 20 nM RECQ1.
(B) Unwinding assays performed at increasing RECQ1 concentrations (filled and open diamonds [⧫◊⋄] indicate 1 nM, filled and open circles [•○] indicate 2.5 nM, filled and open triangles [▴▵] indicate 5 nM, and filled and open squares [□] indicate 10 nM).
(C) Strand annealing assays performed at increasing RECQ1 concentrations (filled and open triangles [▴▵] indicate 10 nM, filled and open squares [□] indicate 20 nM, and filled and open circles [•○] indicate 50 nM).
(1.3 MB TIF)

Figure S3 Transmission Electron Microscopy of Negatively Stained RECQ1 Oligomers Untagged RECQ1 observed in negative stain at 100 kV using a CM100 microscope, alone (A) and in the presence of ssDNA (B). Higher-order oligomers with ring-like appearance are circled, whereas monomers or dimers are boxed. A more homogeneous population of mainly high-order oligomers is observed in the presence of ssDNA. Scale bar represents 200 nm.
(5.9 MB TIF)

Figure S4 Size Exclusion Chromatography Analysis of the K119R Mutant The His_6_-K119R RECQ1 mutant was loaded on the Superdex200 HR 10/30 gel filtration column in the presence or absence of ATPγS. The mutant elutes in two single peaks as observed for wild-type RECQ1. However, the presence of 1 mM ATPγS in the elution buffer does not affect the ratio between the two peaks.
(654 KB TIF)

Figure S5 Competition Experiments with the 30- and 40-bp Forked Duplex Substrates (A) Left panel: time courses of the unwinding reactions with the 30-bp substrate in the presence of 5 nM RECQ1 and 0–100 nM K119R (filled square [] = 0, open left-pointing triangle [◃] = 1.25, filled star [★] = 2.5, open triangle [▵] = 5, filled diamond [⧫] = 10, open circle [○] = 25, filled inverted triangle [▾] = 50, open square [□] = 75, and filled circle [•] = 100 nM). The fraction of ssDNA released as a product of the unwinding reaction is plotted versus time. Right panel: time courses of the unwinding reactions with the 40 bp substrate in the presence of 20 nM RECQ1 and 0–400 nM K119R (filled square [] = 0, open left-pointing triangle [◃] = 5, filled star [★] = 10, open triangle [▵] = 20, filled diamond [⧫] = 40, open circle [○] = 100, filled inverted triangle [▾] = 200, open square [□] = 300, and filled circle [•] = 400 nM)
(B) Plot of the unwinding rates versus the concentration of K119R with the 30-bp (left) and 40-bp (right) substrate. The solid line represents the fit of the experimental data to Equation 1 with *n* = 0.6 ± 0.1.The dashed-dotted, dashed, and dotted lines simulate the expected variation of unwinding rate for *n* =1, 2, or 6.
(1.4 MB TIF)

### Accession Numbers

The following EM maps have been deposited in the EM Data Bank (EMDB) database (http://www.ebi.ac.uk/msd-srv/emsearch/index.html) under the following accession numbers: K119R mutant RECQ1 + ATPγS (EMD-1278), K119R mutant RECQ1 + ssDNA (EMD-1279), tagged wild-type RECQ1+ssDNA (EMD-1277), and untagged wild-type RECQ1+ssDNA (EMD-1276).

## Abbreviations

3D: three-dimensional
bp: base pair
EM: electron microscopy
MSA: multivariate statistical analysis
ssDNA: single-stranded DNA
